# Genomic variations and distinct evolutionary rate of rare alleles in *Arabidopsis thaliana*

**DOI:** 10.1186/s12862-016-0590-7

**Published:** 2016-01-27

**Authors:** Shabana Memon, Xianqing Jia, Longjiang Gu, Xiaohui Zhang

**Affiliations:** School of life Sciences, Nanjing University, Nanjing, 210093 China; Lecturer, Department of Plant Breeding and Genetics, Sindh Agriculture University, Tando Jam, Hyderabad, 70060 Pakistan

**Keywords:** Rare allele, Relative evolutionary rate, Nucleotide polymorphism, Allele frequency

## Abstract

**Background:**

The variation rate in genomic regions associated with different alleles, impacts to distinct evolutionary patterns involving rare alleles. The rare alleles bias towards genome-wide association studies (GWASs), aim to detect different variants at genomic loci associated with single-nucleotide polymorphisms (SNPs) inclined to produce different haplotypes. Here, we sequenced *Arabidopsis thaliana* and compared its coding and non-coding genomic regions with its closest outgroup relative, *Arabidopsis lyrta,* which accounted for the ancestral misinference*.* The use of genome-wide SNPs interpret the genetic architecture of rare alleles in *Arabidopsis thaliana,* elucidating a significant departure from a neutral evolutionary model and the pattern of polymorphisms around a selected locus will exclusively influence natural selection.

**Results:**

We found 23.4 % of the rare alleles existing randomly in the genome. Notably, in our results significant differences (*P* < 0.01) were estimated in the relative rates between rare versus intermediate alleles, between fixed versus non-fixed mutations, and between type I versus type II rare-mutations by using the *χ*^2^-test. However, the rare alleles generating negative values of Tajima’s *D* suggest that they generated under selective sweeps. Relative to polymorphic sites including SNPs, 67.5 % of the fixed mutations were attributed, indicating major contributors to speciation. Substantially, an evolution occurred in the rare allele that was 1.42-times faster than that in a major haplotype.

**Conclusion:**

Our results interpret that rare alleles fits a random occurrence model, indicating that rare alleles occur at any locus in a genome and in any accession in a species. Based on the higher relative rate of derived to ancient mutations and higher average *D*_xy_, we conclude that rare alleles evolve faster than the higher frequency alleles. The rapid evolution of rare alleles indicates that they must have been newly generated with fixed mutations, compared with the other alleles. Eventually, PCR and sequencing results, in the flanking regions of rare allele loci confirm that they are of short extension, indicating the absence of a genome-wide pattern for a rare haplotype. The indel-associated model for rare alleles assumes that indel-associated mutations only occur in an indel heterozygote.

**Electronic supplementary material:**

The online version of this article (doi:10.1186/s12862-016-0590-7) contains supplementary material, which is available to authorized users.

## Background

The extensive studies based on whole genome sequencing of species, apparently involving polymorphism in the same species and understanding the pattern of genomic variation at a particular locus profoundly exploits the sophisticated functional gene evidence in population genetics [1–3]. The genome-wide association studies (GWAS) have detected variants at genomic loci which are connected with complex traits in a population [4]. Collectively, most of the species have varying frequent genetic diversity including rare alleles, which seem to disappear in a large population due to deleterious mutation [5]. Rare variants including polymorphisms with low allelic frequency are able to determine the architecture of genetic complex traits [6–8], which are distributed in two types of substitutions: type I rare substitution, which disperses randomly in different haplotypes and type II rare substitution or ‘rare allele’, which gathers together in one haplotype. Here, the haplotype means a set of substitutions that tend to always occur together on one chromosome. Whereas, the common polymorphisms are capable to describe only a small proportion of genetic variation in a particular trait [8]. According to an exception, ancient allele may be rare as it diminish from the population and takes many generations to give rise to a reasonable allelic frequency. Whereas, a common allele may be recent which emerge from a rapid population expansion [5]. Rare variants seem to have dominant effects containing haplotypes with functional alleles [9]. They relatively appear to have a high level of singleton replacements [10]. For instance, the *AP1* locus contributes 73 of the 92 nucleotide polymorphisms in only a single allele and is evident to have more than one functional characteristic [10].

Broadly, GWAS emphasize the variation occurred in the natural population and the use of genome-wide SNPs in genome wide association studies provides not only the discovery of truly *de novo* candidate gene, but also interprets the comprehensive view of genetic architecture of the traits [11]. The genome-wide surveys of nucleotide polymorphisms in *Arabidopsis thaliana* have elucidated a significant departure from a neutral evolutionary model, due, in part, to an excess of rare variants [7, 12]. Substantially, the pattern of polymorphisms around a selected locus exclusively influences natural selection [13].

Rare alleles are considered as polymorphic alleles having <1 % frequency [5]. However, to detect the functional rare alleles the frequency depends upon the sample size and those rare variants having sample size limited are in ineffectual. It has been reported that in some cases rare variants having minor allele frequency (MAF) <5 %, often exclude single nucleotide polymorphisms from the genome wide association studies and have determine the genetics of complex traits [3, 8]. Low frequency variants are recent in origin exhibiting population differentiation and are potential for functional mutations pertaining under weak purifying selection [14]. However, when the population size is low the deleterious variants are accumulated, as purifying selection is ineffective in eliminating these variants as the population size decreases [15]. Low frequency variants are more essential to elucidate the genetic architecture and stratification occurring in genomes, uncovering the complexity of a population [6].

Understanding, the various mechanisms involved in the identification of putative rare variants and maintenance of a genome-wide excess of these variants essential consideration of genetic variation in living organisms are required [16]. The population structure can deviate the genome-wide pattern of variation from standard neutral models [12]. Mostly the neutral expectations are derived from the polymorphism of functionally and putative neutral sites [17]. Hence, critical advances in relevant area are feasible on three basic hypotheses proposed to explain beyond a neutral model. First, it can be expected that low allelic frequency might have arisen from a recent selective sweep of an advantageous allele at a locus [18–21], or from fixation of locally occurring polymorphisms by selection [7]. Generally, in this process, the hitchhiking concept is detected in which advantageous mutations with closely linked beneficial alleles exist and the potential sites are targeted by selective sweeps and neutral mutations [22–25], producing new recombination and polymorphism. The targeted sites with strong selective sweep depend upon the size of the affected region [24]. The power of selection with these haplotypes is expected to contain low frequency rate and extend several hundred kb long [26].

Since low frequency variants describe the etiology of complex traits, so secondly, it is critical to elucidate the architecture of population, its history, structure and dynamics in the form of excess linked SNPs (single nucleotide polymorphisms), estimating at both low and high frequency [6, 7, 26, 27]. For instance, a recent expansion combined with the metapopulation behavior of *Arabidopsis thaliana*, might explain the excess of rare alleles at the *TGG1* locus [26]. The population-associated hypotheses generally predict a genome-wide pattern of rare alleles and an equal evolutionary rate between rare allele and major haplotype.

Finally, to determine the mutation rate variation [28, 29] implicated in the level of nucleotide diversity between the two haplotypes mentioned above, the striking trends including the association between indel (insertion/deletion) mutations and nucleotide substitutions[28, 30] and an indel-associated suppression of crossover (and recombination) during meiosis [31], favor the hypothesis involving a local increase of nucleotide substitution rate. However, these indel-associated mechanisms lead to more accumulated substitutions linked to the indel site between the indel and non-indel haplotypes. Moreover, the increased substitution appeared around indels are said to be indel-lineage specific [30]. Thus, an indel (insertion and deletion mutations) contributing genetic divergence in a haplotype will implicate a higher evolutionary pattern existing in such alleles [32]. Consequently, the frequency of newly occurred indel is low among populations and an indel haplotype should rather be a rare allele [30]. Therefore, the small indel mutation rate is strongly dependent on short tandem repeats and are functionally important for polymorphism [33, 34]. Also, such rare alleles are expected to extend to a short genetic distance, due to the effect associated with indels only in the regions several hundred-bp from indels [30].

Several GWAS studies including high SNP density have been studied in Arabidopsis, rice and maize [3]. However, the array-based genotyping approach using the three different models as mentioned above will deploy the expected patterns of rare alleles. Therefore, an investigation on the genomic distribution, evolutionary rate and the extended length of rare alleles was distinguished, elucidating the mechanisms involved to generate such alleles and how these rare alleles evolved. Presently, we used a genome-wide polymorphic dataset of *Arabidopsis thaliana* to survey the rare allele patterns. We also utilized *Arabidopsis lyrata*, in a sister clade to *A. thaliana*, as a reference to examine evolutionary rates between rare and major haplotype. Eventually, we discovered distinct genomic distributions and evolutionary rates for rare alleles. Our results strongly suggest that higher local rates of substitutions among different alleles can generate the excess of divergent variants at low as well as high frequencies.

## Methods

### Genome sequences and alignments

The aligned sequences of 96 *Arabidopsis thaliana* accessions were downloaded from http://1001genomes.org/accessions.html for each of 1214 loci, representing one locus every 100 kb and 574.3 bp per locus on average [12]. Eighty-four loci were excluded because they either contained fewer than 60 accessions or were shorter than 400 bp. The outgroup sequence, *A. lyrata*, was obtained from http://genome.jgi-psf.org/Araly1/Araly1.download.ftp.html, which comprised 206.7 Mbp sequences with about 8-fold genome coverage. To locate *A. lyrata* orthologs for the remaining 1130 *A. thaliana* loci, the accession of the “Columbia” sequence from each of these loci was used for a local BLASTN search against *A. lyrata* sequences (using an e value threshold <1E-3): this procedure yielded 1118 ortholog sequences. After excluding the loci for which identity was <0.85, only 939 *A. thaliana* loci were found to be aligned with *A. lyrata*.

### Distinct rare alleles at a locus

**Fig. 1:**
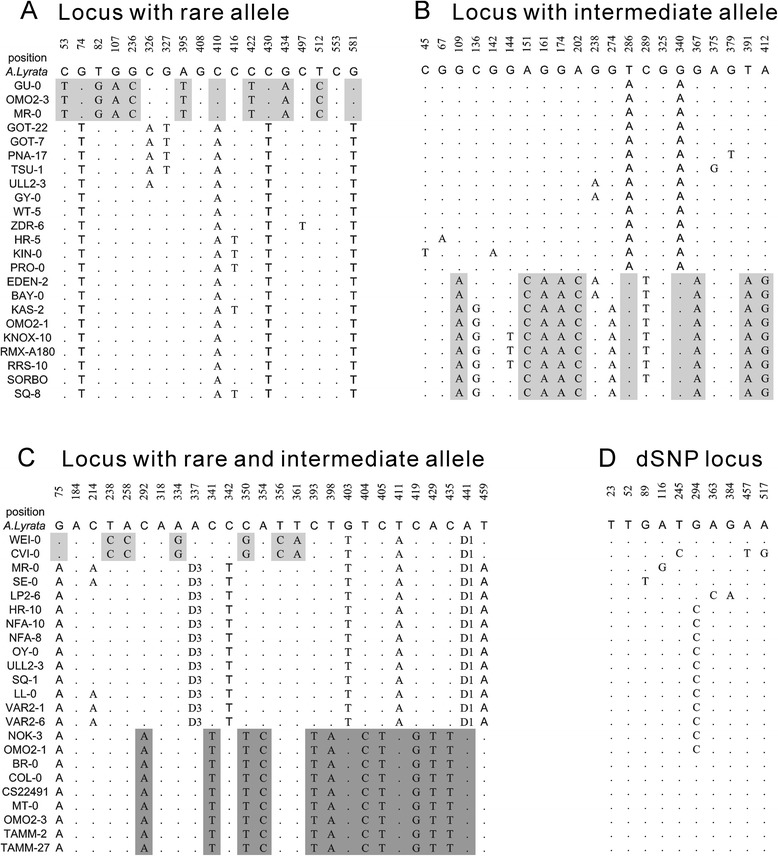
Examples of rare allele, intermediate allele in a gSNP locus and complicated and dSNP locus. Dot indicates identical nucleotide compared to ortholog (*A. lyrata* sequence relative to the alignment of *A. thaliana*). The numbers above the ortholog sequence denote nucleotide positions in the sequence. Fixed sites between haplotypes at rare or intermediate alleles are shown in grey shadow. **a** An example of a rare haplotype in gSNP locus is located at 18433043 (chr. 1). **b** An intermediate allele is shown in gSNP locus which is at 24321799 (chr. 1). **c** An example used as a complex locus that holds two distinct haplotypes of both a rare allele (light grey shadow) and an intermediate allele (dark grey shadow) is located at 4826763 (chr. 1). **d** An example of a dSNP locus is at 10095832 (chr. 1). Only part of the accessions within each haplotype is displayed in all examples

We defined a distinct haplotype at a locus as having at least 5 fixed nucleotide substitutions (or fixed mutations). Because the average length per locus was approximately 500 bp, the fixed nucleotide diversity between haplotypes was ~1 %. This criterion was chosen on the basis of clearly identified haplotypes in a sequence alignment (*e.g*., Fig. 1a). Considering the low average diversity (0.44 %) in the 939 loci, and the small proportion of fixed mutations in the total substitutions (0.406 = 5625/13859), this criterion was generally enough to discriminate different haplotypes. Also, the neutral simulation results [35] showed that the distinct haplotype was not from random samples (*P* < 0.05; Additional file 1: Figure S1).

A distinct haplotype could contain one or more accessions. When a distinct haplotype contains 9 or less accessions (<10 % frequency in total 96 accessions), this haplotype is defined as a type II rare allele. Accordingly, a distinct haplotype, with accession frequency ranging from 10 % to <50 %, is named as an intermediate allele, and the major allele is defined as the haplotype which contains the majority of accessions. At a locus, there could be 1, 2 (Fig. 1a-b) or >2 distinct haplotypes (Fig. 1c). When a locus has only one haplotype, it must be a major allele (Fig. 1d). At such locus, the SNPs are randomly (or near randomly) distributed. Because the randomly-distributed SNPs looks as dispersed in any of 96 accessions, these SNPs are defined as dSNPs and the loci with only major allele are denoted as dSNP loci (Fig. 1d). When a locus has two or more distinct haplotypes, one of them must be a major allele and other(s) be a rare or intermediate allele. Because the fixed substitutions between these alleles look as grouped SNPs, these loci are defined as gSNP loci, the fixed substitutions as gSNPs, and the non-fixed substitutions as nfSNPs (Fig. 1a-c). Both nfSNPs and dSNPs are essentially the SNPs randomly-distributed within a haplotype, so they are named as rSNPs. All categories of rSNPs can be further divided into low-frequency (<10 %; nfSNPs_<10%_ or dSNPs_<10%_, respectively) and intermediate-frequency (10–50 %; nfSNPs_≥10%_ or dSNPs_≥10%_, respectively) classes. nfSNPs_<10%_ and dSNPs_<10%_ were type I rare substitutions. In summary, the SNPs included gSNPs, nfSNPs and dSNPs.

When using *A. lyrata* as the outgroup sequence, any of the gSNPs, nfSNPs or dSNPs could be inferred as new (derived) or ancient mutations. When a mutation site between rare/intermediate and major alleles had different nucleotides from those in *A. lyrata* sequence, this site was excluded, since the mutation direction could not be defined by the outgroup. For each category of haplotypes or SNPs, the relative rate of derived to ancient mutations (hereafter referred to as “relative rate”) reflects the relative evolutionary speed of the allele. For example, if the number of derived mutations in a rare-allele is greater than the ones in a major allele, this rate (>1) indicates that the rare-allele evolves faster, relative to the major haplotype.

Similarly, the fixed indel mutations between haplotypes can be also inferred as derived or ancient mutations. Those indels with different sizes between different haplotypes are excluded in our analysis. The relative rate for indels is calculated between rare and major alleles.

By the above criteria, if more than one distinct haplotypes could be identified at a locus (Fig. 1c), it was denoted as multiple-haplotype or complex locus.

### Genotyping and sequencing

To detect the extension of rare alleles, nine loci were selected from the gSNP loci randomly. PCR amplification and sequencing were carried out in the flanking regions around each of the selected rare-allele loci in 10–12 accessions using chromosome walking method, until a 500-bp region, in which the specific pattern for a rare allele was disappeared, was obtained. The PCR Primers were designed from the Col sequence, and sequencing reactions were run on an ABI 3100-Avant automated sequencer.

## Results

### Identification of rare alleles and the polymorphism between alleles

A total of 939 gene loci were chosen for our study according to our criteria (≥60 accessions, containing ≥ 400 bp, with only one ortholog in *A. lyrata* for each locus, as in Materials and Methods). Out of 939 gene accessions, 220 gene loci has Type II rare variants, contributing 23.4 % of the loci (Fig. 1a), indicating that they are common polymorphisms in *A. thaliana*. Whereas, the intermediate-frequency haplotypes accounted for 22.4 % having a total of 211 gene loci (Fig. 1b, Table 1). From the remaining proportion of 939 loci, 40.3 % of loci analyzed (378 out of 939 loci) contained either rare variants, intermediate ones, or both of them, with 11.0 % (103 loci) holding more than two distinct alleles (Fig. 1c).

**Table 1:** Nucleotide mutations at 939 loci of *A. thaliana* with *A. lyrata* as outgroup

Items	Loci	Haplotype	Total	Derived	Ancient	Ratio
gSNP	378	555	5625	2722	2218	1.25
Rare (typeII)	220	282	2881	1497	1052	1.42
Intermediate	211	273	2744	1275	1166	1.09
nfSNP	378	/	3833	3056	388	7.88
Rare (typeI)		/	2935	2453	187	13.12
Intermediate		/	898	603	201	3.00
dSNP	561	/	4401	3519	491	7.17
Rare (typeI)		/	3442	2883	261	11.05
Intermediate		/	959	636	230	2.77
Total/Ave	939	555	13859	9347	3097	3.02

Haplotypes represent the number of different rare and/or intermediate haplotypes in gSNPs loci. Total = total number of SNPs in a certain allele. Derived = newly occurring mutation; Ancient = ancient mutations; Ratio = the relative rates of derived to ancient mutations

**Fig. 2:**
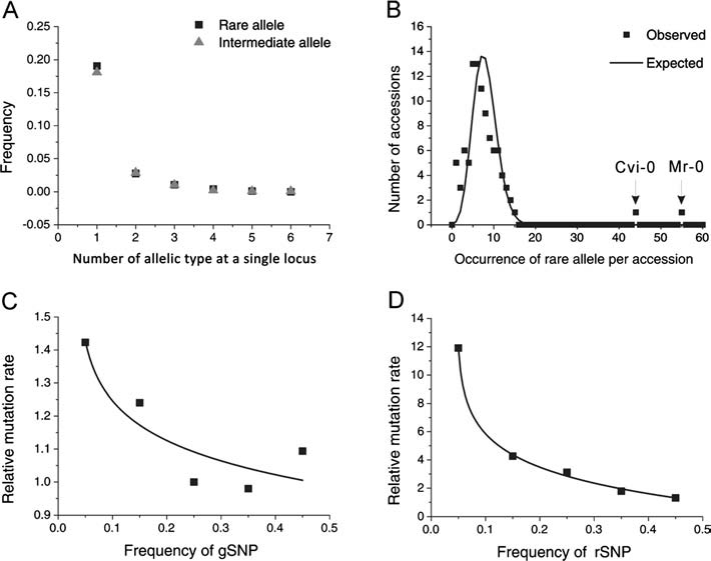
Frequency distribution of rare alleles and nucleotide substitutions. **a** Observed frequency for the loci with different types of rare and intermediate alleles separately. **b** Distribution of rare alleles in 96 accessions at 939 loci. The x-axis represents the total number of occurrence of rare alleles per accession and the y-axis denotes the total number of accessions which contain the same number of occurrence. The expected number is estimated under the random occurrence model with all the accessions. **c**- **d** The relationship between relative rates of derived to ancient mutations and frequency of 5625 gSNP sites, the fixed mutations (**c**), and 8234 rSNPs sites (including nfSNP and dSNP) (**d**)

The Type II rare variants containing 220 gene loci, exhibited 282 rare alleles which correspond to produce more than one rare haplotypes. Relevant to these loci, 179 genes (19.06 %, F_1_) exhibited a single type of rare allele, and the remaining 26 (2.77 %, F_2_), 10 (1.06 %, F_3_), 4 (0.43 %, F_4_) and 1 (0.11 %, F_5_) loci contained two, three, four and five types of rare alleles, respectively (Fig. 2a). Accordingly, the random occurrence model was used to estimate the expected frequencies for the loci contributing different types of rare alleles and also estimated for those loci containing the intermediate frequency haplotypes. The random occurrence model was calculated by the formula below [36].

$$ \mathrm{E}\kern0.5em {\left[{\mathrm{F}}_{\mathrm{i}}\right]}_{\left(\mathrm{i} = 2,\ 3,\ 4\ \mathrm{or}\ 5\right)} = {{\mathrm{F}}_1}^{\mathrm{i}}, $$

Where E [F_2_] = 0.1906^2^ = 3.63 %, E [F_3_] = 0.1906^3^ = 0.69 %, E [F_4_] = 0.1906^4^ = 0.13 %, and E [F_5_] = 0.1906^5^ = 0.03 %. Notably, no significant difference was estimated between observed and expected frequencies (*χ*^2^ = 3.23; *P* > 0.05), which suggested that at a single locus two or more different rare allelic types occurred independently.

The allelic frequency distribution relative to polymorphic sites indicating skews toward SNPs with low frequency exhibit significant negative values of Tajima’s D and can result from positive selection, while positive values of Tajima’s D statistic arise from an excess of loci with intermediate frequency haplotypes [12, 34, 37–40]. Substantially, the expected average result of Tajima’s *D* statistic [41] for the rare allele loci seem to be significantly lower (−1.24) than that of the other loci (−0.69). From the statistical results it was observed that 100 % of the 22 loci with Tajima’s *D* < −2.2, and 81.4 % of the 59 loci with Tajima’s *D* < −2, were rare-allele loci, while 92.7 % of the 96 loci with Tajima’s *D* > 1.0 contained no rare alleles (Additional file 1: Figure S2). The results elucidates that the rare alleles generating negative values of Tajima’s *D* suggest that they generated under selective sweeps must be mainly due to the identified rare-alleles, predicted according to the criterion used for the identification of such locus, since no such high frequency derived alleles was observed in the simulations. Under recurrent selection sweep deleterious alleles segregate at low frequency corresponding to the negative Tajima’s *D* [42].

Furthermore, we compared the observed and expected numbers of polymorphic alleles in which rare alleles occurred in all accessions, predicted from the frequency distribution. Two accessions, Mr-0 and Cvi-0 seem to likelihood contain higher rare alleles and deviated from the other accessions (Fig. 2b). These results are consistent with random occurrence model representing the frequency distribution of occurrence of rare alleles among all accessions along with these two accessions. Beyond the criteria, considering both the rare and the intermediate alleles, the frequency distributions among accessions are quite satisfactory with the random occurrence model (Additional file 1: Figure S3). This result suggests that a rare allele could occur randomly in every accession with an equal possibility in a population.

### Patterns of mutation rate variation influencing evolution

Mutation rate heterogeneity can be detected by multiple scales [30]. Exclusively, we investigated the rate of Mutations occurred in a rare allele (or intermediate allele) *vs*. a major allele, which can be differentiated by a reference sequence (see Materials and Methods for definition) and could be used to determine the relative evolutionary outcomes, associated between these haplotypes. The mutation rates observed were distinct among various haplotypes (Table 1). For instance, 2549 fixed-segregating sites from a total of 2881 between rare and major alleles could be clearly discriminated as mutations that occurred either in rare (1497) or in major alleles (1052, the ancient mutations). The other 332 fixed sites could not be distinguished, and were excluded for further analysis, because of insertions/deletions found between *A. lyrata* and *A. thaliana*, or because *A. lyrata* sequences differed from both rare and major haplotypes at these sites. With outgroup sequences (see Fig. 1) we obtained a relative rate of 1.42 (=1497/1052) in rare *vs.* major allele, indicating an evolution in the rare allele that was 1.42-times faster than that in a major haplotype.

In particular, the type I rare variants have an extraordinarily high relative rate of mutations (13.12, Table 1), indicating that almost all rare variants were generated by derived mutations. Similarly, relative rates were also calculated for the intermediate *vs.* the major allele (1.09). Notably, Significant differences (*P* < 0.01) were estimated in the relative rates between rare versus intermediate alleles, between fixed versus non-fixed mutations, and between type I versus type II rare-mutations by using the *χ*^2^-test.

Further, comparisons of the incidence of indels reveal that the relative rate of SNP is significantly largely lower than obtaining higher frequency SNPs. Figure 2c-d demonstrates that the relative rates of both gSNPs and rSNPs decrease as their frequencies increase. Under neutral evolution model, the number of mutant (new) alleles should decay exponentially with the increasing of the frequency in populations [43]. The decay curves in Fig. 2c-d suggest that the relative rates vary consistently with neutral expectation of allelic frequencies.

When considering fixed insertion/deletion (Indel) in the rare allele and major allele, we found that the derived indel mutations in rare alleles (182) was higher than ancient ones (78) in major alleles. The much larger relative rate for indels between these two types of alleles (182/78 = 2.33), compared with 1.42 for SNPs, suggests that indels decayed more drastic as their frequencies increase. Interestingly, while distinguishing between the mutation rates of indel- and non-indel loci out of the 939 loci analyzed, we observed 2.17 times higher in indel than that in non-indel loci.

### Allele frequency distribution

According to a standard population genetics model, the allelic frequency is expected to fit a neutral distribution [41]. Normally, only minor allele frequencies (MAFs) are available for such analysis, e.g., in *Arabidopsis* [12]. Because of the availability of the outgroup sequence *A. lyrata*, a reference can be used to infer the unfolded site frequency spectrum [44], and to detect the excess of rare variants.

**Fig. 3:**
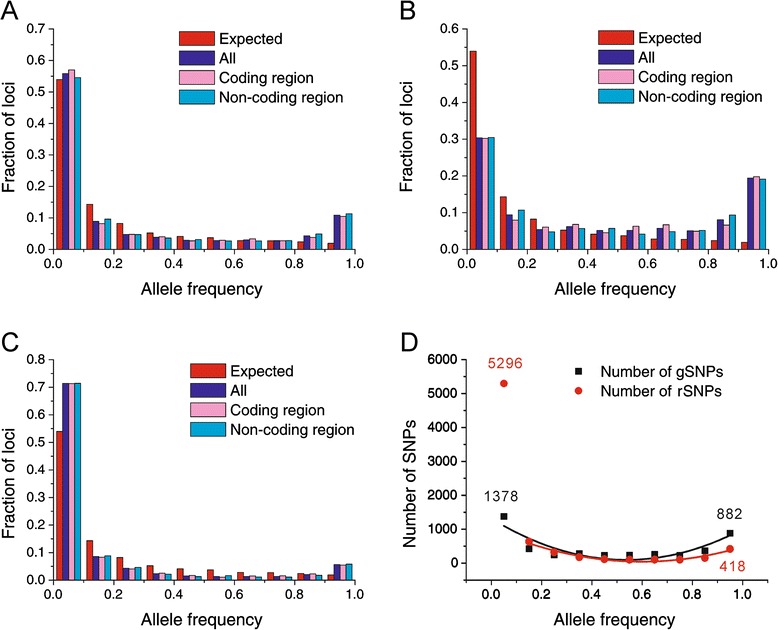
The allelic frequency distribution of SNPs occurring in all regions, coding and non-coding regions with *A. lyrata* as an outgroup sequence in all of the 939 loci: (**a**) all SNP; (**b**) gSNP sites; (**c**) rSNPs sites (including nfSNPs and dSNPs); (**d**) The number distribution of gSNPs with the fitting formula y = 1315.0 – 4535.6x + 4230.7x^2^ and dispersed SNPs with the fitting formula y = 1012.8-3221.7x + 2695.4x^2^. The expected allele frequency distribution with an outgroup sequence under a standard constant-size population genetics model in **a**, **b** and **c** is given by (1/i)/∑n-1j = 1 1/j, for i mutants per site, where n is the sample size (Ewens [43])

The excess of low frequency SNPs, observed in a polymorphism study [12], was present when referred by MAFs (Additional file 1: Figure S4) and by outgroup sequences (Fig. 3a), suggesting that these mutations, referred by *A. lyrata*, had a similar distribution pattern. To measure the surplus of mutations, we defined the frequency excess as the observed frequency/the expected frequency – 1, which was 0.20 and 0.034 for the mutations at the frequency < 0.1, when referred by MAFs and by outgroup sequences, respectively. These values indicated that there was only a slight excess of rare variants when referred by an outgroup sequence. However, we found the frequency excess to be 4.64 (*P* < 0.001) for SNPs at the high frequency range (0.9 - <1; Fig. 3a); this value was 23.2 (or 136 = 4.64/0.034) times more than that for the excess of rare variants. Detailed distributions of gSNPs and rSNPs (including nfSNPs and dSNPs) (Fig. 3b–c) further demonstrated that the high-frequency excess was due in large part to the excess of gSNPs (the frequency excess = 0.194/0.019 -1 = 9.09; *P* < 0.001), while the low-frequency excess was largely contributed by nfSNPs and dSNPs (excess = 0.32; *P* < 0.001). These results strongly suggest that the characteristics of gSNPs are quite different from those of rSNPs, and that the mechanisms for their occurrence and maintenance may be different.

### Contribution of gSNPs to the divergence between species

To characterize gSNPs and rSNPs, we calculated their contributions to the divergence between species. The full range of frequency distributions in SNPs provided a unique opportunity to examine the contribution of fixed mutations in gSNPs and rSNPs to the divergence between species. In Fig. 3d, the number distributions of SNPs show clearly that the rSNPs are 3.84 (5296/1378) times more than gSNPs in the low frequency range (<0.1); however, they are only 47.4 % (418/882) of gSNPs in the high frequency range (>0.9). If the ratio of high to low frequency SNPs is used to measure the fixation probability, it is 64.0 % (882/1378) for gSNPs but only 7.9 % (418/5296) for rSNPs. These results suggest that the dSNPs and nfSNPs were generated much more frequently; however, they were also lost more quickly before fixation. On the other hand, gSNPs were generated much less frequently, but they could stay in populations more than 8 times longer (64.0 % versus 7.9 %), and therefore they became a major type of polymorphisms.

Furthermore, a quadratic curve best fits the observed distributions of both types of SNPs (r = 0.89 and r = 0.97, respectively, and *P* < 0.001 for both in Fig. 3d). Thus, based on fitting to the curves, the fixed SNPs (when the frequency =1) were deduced to be 1010 and 486 for gSNPs and rSNPs, respectively. The fixed mutations should determine the level of the divergence between species. Our analysis demonstrates that 67.5 % of the fixed mutations could be attributed to gSNPs, indicating that these mutations were major contributors to speciation.

The nucleotide divergence (*D*_xy_) between *A. thaliana* and *A. lyrata* further revealed the different contributions of the gSNPs and the rSNPs to the divergence between species. A significantly higher *D*_xy_ (*P* < 0.01) was observed for rare and intermediate haplotypes than for major alleles at either gSNP loci or dSNP loci (Additional file 1: Figure S5). This trend was present in both coding and non-coding regions, suggesting that evolutionary rates between the loci with and without gSNPs or between the haplotypes with more and less gSNPs are different. The gSNP loci, or the haplotype with more gSNPs, apparently evolve faster.

### Distribution of gSNPs and rSNPs in coding regions

**Fig. 4:**
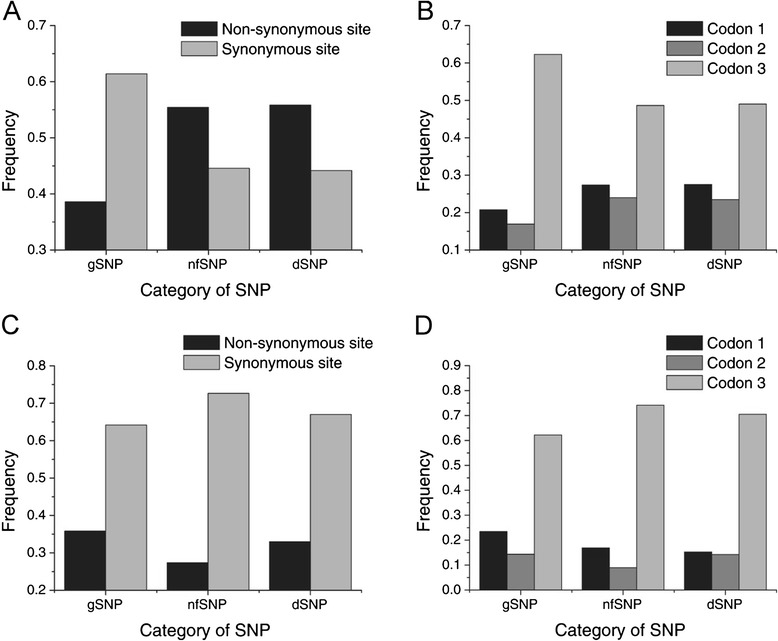
Characteristics of gSNP, nfSNP and dSNP in the coding region: (**a**) Comparison of the distribution of non-synonymous and synonymous SNPs in derived mutations (allele frequency <0.5) of gSNP, nfSNP, and dSNP sites, respectively. **b** Distribution of SNPs occurred at the first, second and third position of a codon in derived mutations of gSNP, nfSNP and dSNP sites, respectively. **c** Comparison of non-synonymous and synonymous SNPs distribution in ancient mutations (allele frequency ≥0.5) of gSNP, nfSNP and dSNP sites, respectively. **d** Distribution of SNPs occurred at the first, second and third position of a codon in ancient mutations of GNP, nfSNP and dSNP sites, respectively, in which the total frequency is 1 for each category of SNPs in **a**-**d**

We also found that the distributions of gSNPs, nfSNPs and dSNPs were quite different in terms of the positions of codons, and in the synonymous and non-synonymous substitutions. In derived mutations from gSNPs to rSNPs (Fig. 4a-b), a clear decrease was noted in the proportion of synonymous mutations, and in the third-position mutations of a codon, while a clear increase was observed in the proportion of non-synonymous mutations and in first- (or second-) position mutations, implying that gSNPs are more detrimental to gene integrity than nfSNPs and dSNPs. In contrast, the opposite type of distribution was observed for ancient mutations (Fig. 4c-d), indicating that as time went on, a greater proportion of gSNPs were selectively maintained. Thus, gSNPs contribute more heavily to the variations in amino acids.

Unexpectedly, the transitions/transversions ratios for gSNPs and for rSNPs were significantly different: 1.102 *vs.* 1.249 (*P* < 0.05) and 1.293 *vs.* 1.504 (*P* < 0.05) for derived and ancient substitutions, respectively. The ratio for rSNPs was higher than for gSNPs, or was higher for ancient substitutions than for derived mutations. These findings suggest that different mechanisms underline the generation or maintenance of different types of SNPs. However, the mechanisms themselves remain to be determined.

### Extension of rare alleles

A study of the length of rare alleles may provide evidence to enable discrimination between different hypotheses about how these alleles were generated. For example, substantial extension of a rare allele could support the ‘selective sweep’ or ‘introgression’ hypotheses, and in particular may support the theory of ‘recent population expansion’. To estimate the extension of a rare allele, we sequenced the flanking regions of nine loci, which were randomly selected from loci containing type II rare-allele(s). About 10–12 accessions were sequenced to detect the presence or absence of a rare allele in their 5′- and 3′-flanking regions. Extension of the rare allele was determined by the disappearance of the rare polymorphic pattern in a >500 bp sequence of each of two flanking regions (Additional file 1: Figure S6). The 10–12 accessions at the nine loci contained 10 patterns of rare alleles. Nine out of the 10 patterns were found to disappear out of a distance of 521–2763 bp (average 1293 bp), while the remaining one extended to >6 kb (Additional file 1: Figure S6, the locus 4–9869625). These results show that the extension of rare alleles is generally short, indicating that the factor(s) which generates the rare alleles is locally-associated.

## Discussion

### Characteristics of rare alleles

The hypothetical concept of using distantly related species *Arabidopsis lyrata* as an outgroup is to polarize the substitutions along each lineage and could result in a misinference of the ancestral state, which would lead to an excess of high frequency of the derived state [44, 45] . Morton *et al*. [44] analyzed the unfolded site frequency spectra in *A. thaliana* with *A. lyrata* as reference, and accounted for the ancestral misinference with use of the method of Baudry and Depaulis [46]. They found that the skew toward sites with a high frequency of the derived state could not be explained by ancestral misinference. Thus our strategy of using an outgroup sequence to characterize the rare alleles was appropriate and five phenomena were observed.

First, by using conserved criteria (e.g., ≥ 5 fixed mutations), the rare alleles were revealed to be common in *A. thaliana*. Our data interpreted that 23.4 % of the loci contained an average of 1.28 rare alleles and 100 % of the accessions with a 0.8 % (759/939/96) probability of being a rare allele per accession at any locus. The occurrence of rare alleles fits a random occurrence model. This indicates that the rare alleles could occur at any locus in a genome and in any accession in a species. These characteristics provide a basis for analyzing rare alleles as neutrally occurring polymorphisms.

Second, based on the higher relative rate of derived to ancient mutations and higher average *D*_xy_, we conclude that rare alleles evolve faster than the higher frequency alleles, the major alleles or sequences at the loci without the grouped nucleotide polymorphisms. This characteristic is understandable because more mutations occur in rare alleles rather than in other alleles or in the dSNP loci. Consequently, a higher *D*_xy_ in rare alleles are expected. A rare allele is considered to be ancient as it is depleted from the population and generally consists of long haplotypes [5]. The relative rate essentially reflects the relative evolutionary speed between rare- and the other alleles.

Third, the rapid evolution of rare alleles indicates that they must have been newly generated, compared with the other alleles. Newly generated fixed mutations in rare alleles account for only a fraction of the all mutations, compared with the rSNPs. However, they can stay in populations much longer, and eventually contribute greatly to the nucleotide variation among accessions and to the divergence between species.

Fourth, the extension of a rare allele is generally short. Our PCR and sequencing results in the flanking regions of rare allele loci confirm that about 90 % of rare alleles are short (about 1.3 kb), although a rare allele that is >6-kb has been identified. The short extension of rare alleles indicates the absence of a genome-wide pattern for a rare haplotype. The length of extension in a rare allele may be associated with its age and its level of fixed mutations, but we were unable to address these issues without sufficient sequences around the rare alleles.

Finally, the rare alleles are likely generated by the same factors which lead to the higher frequency alleles. With the exception of their frequencies, which are defined to be, rare alleles and higher frequency alleles have the same characteristics. Indeed, the differences in frequency suggest unique relative rates; however, such differences are much smaller among them than the differences between them and the sequences at the dSNP loci. Overall, the relative rates, the quantity of mutations at the low frequency, the possibility of loss or fixation, and the duration of time that they remain in the population are all quite different between gSNPs and rSNPs. Thus these characteristics of rare alleles or gSNPs are critical for understanding their origin and maintenance, the pattern of nucleotide polymorphisms and their differentiation within and between species.

### Occurrence of rare alleles

Two hypotheses are often used to explain the generation and maintenance of rare alleles. First, a recent selective sweep of an advantageous allele at a locus is often used to explain the occurrence of rare alleles [10, 19, 20]. In general, a selective sweep involves a target gene which is under positive selection, resulting in hitchhiking effect of a long stretch of sequence that generates a long extension of nucleotide signature and young fixed mutations between rare and major alleles. Commonly occurring rare alleles and their short extensions are not consistent with these expectations. Particularly, the value (1.42) of relative rate in rare alleles shows that the mutations are not very young (e.g., compared to the value of 7.48 for non-fixed mutations). Although the selective sweep may generate rare alleles in some loci, it cannot be the general factor that directs the occurrence of such alleles. Given the random distribution of rare alleles in accessions and loci, the assumptions based on natural selection are not viable.

Second, the other major hypothesis is that rare alleles can be generated by a specific population history, structure or dynamics. There is extensive work showing population structure in *Arabidopsis* species [12]. Sequence exchanges among those structured populations could generate some of phenomena observed in this study, e.g., the random occurrence, the short of genome-wide pattern and/or the short extension. More frequent occurrence of rare-alleles, revealed in the accessions Mr-0 and Cvi-0 at the 939 loci analyzed, could also support these hypotheses. On the other hand, some phenomena observed seem incompatible with these assumptions. Theoretically, a recent population expansion, a bottleneck effect, or an admixture from previously separated subpopulations can generate rare alleles. In all these scenarios, the fixed mutations between rare and major alleles should be the substitutions that have accumulated as a result of geographic isolation. Comparisons of these substitutions between a rare accession and an accession from major haplotypes should yield a relative rate that is close to 1, not evolving particularly fast in a geographic region. In addition, the fixed mutations generated by geographic isolation should extend as an accession-specific or genome-wide pattern, when sequence exchanges are restrained such as in the selfing species. In this study, the relative rates in the rare alleles, including Mr-0 and Cvi-0, are observed to be >1, indicating some level of different evolutions from geographic isolation. Also, no genome-wide pattern (such as consistent presence in rare alleles at different loci) could be detected from any of 96 accessions. Considering all these results, population-related hypotheses could in part but not fully explain the generation and maintenance of rare alleles.

### Indel-associated model for the occurrence of rare alleles

Mutations can be generated at a relatively high rate around an indel [30] and haplotypes can be differentiated into indel and non-indel types, due to the locally suppressed recombination [31]. Mutations linked to the indel site could accumulate over time between the rare and major haplotypes. These mechanisms would create rare alleles that should be primarily affected by mutation and neutral drift. Several features revealed by our analysis discussed below support the indel-associated model for the occurrence of rare alleles.

First, the higher mutation rate in indel loci (about 2.17-times higher than non-indel regions) indicates that the high level of nucleotide diversity between rare and major alleles could be generated in a much shorter period of time if indel(s) occurred between them. This could explain why so many fixed mutations accumulate between them, compared with the much fewer nfSNPs. The higher mutation rate around indels and the locally suppressed recombination could explain why the fixed mutations seem to be generated in a relative short time, as indicated by the higher relative mutation rate.

Second, this model assumes that indel-associated mutations only occur in an indel heterozygote (but not a homozygote), and that the total number of indel-associated mutations occurs equally on both the indel and the non-indel alleles in indel heterozygotes. All indels begin their existence at low frequency, and alleles with derived indels would more likely to be in an indel-heterozygote in a random mating population. Rare alleles are often present in heterozygous condition in randomly mating populations [47]. In contrast, the ancestral allele (normally the non-indel allele or the major allele) would more likely to be in a non-mutation-inducing homozygote when the indel is rare. Therefore, the rare allele is expected to have more derived indels, and the number of substitutions for rare allele accessions is different in these individuals from that of major alleles, because the frequencies of the rare accessions at a locus among populations are different. This is why the relative rate is dependent on the frequency of rare alleles that we observed: the lower the frequency, the larger the relative rate. We also found that, when referred by *A. lyrata*, the rare alleles had a higher proportion of newly occurring indels than that of major alleles (182 *vs.* 78).

Third, the indel-associated mutations only occur in nearby regions, which are about 1 kb from the indel [30], indicating that the extension of rare alleles is not very long, except for the regions with more indels between rare and major alleles. The short extension of rare alleles observed suggests that they are generated by locally and randomly existing factor(s). The indels are factors that are consistent with these requirements.

Finally, mutations between indel and non-indel alleles may be easy to maintain, because recombination is found to be restrained around indels. For example, during meiosis insertions reduce the occurrence of crossovers dramatically in their surrounding regions [31] indicating a reduced frequency of recombination around indels. Reduced recombination could act as a genetic isolation between indel and non-indel allele, which could maintain the observed excess of fixed mutations between rare and major alleles.

## Conclusion

In the scenario, of genetic association of rare variants in *Arabidopsis thaliana,* using distantly related species *Arabidopsis lyrata* influenced a misinference of the ancestral state. The rare alleles discriminated a higher likelihood of being generated and maintained constantly, than would be expected with a neutral model. Therefore, rare alleles evolve faster to the stage of higher frequency haplotypes, and can be fixed eventually. Consequently, there were genome-wide excesses of rare alleles, due to higher mutation rates or reduced recombination rates, and also because of variation (polymorphisms) between genomic regions, where some of them contained more gSNPs as compared to others. The mechanisms to generate rare alleles and how rare alleles evolve,which cannot be interpreted by a standard neutral model [7, 12], can be better elucidated by this scenario. Further, comparisons of indel-associated occurrence model of rare alleles could add another explanation for the polymorphisms as observed previously.

## Additional file

Additional file 1: Figure S1. Results of coalescent simulations. **Figure S2**. The distribution of Tajima’s D statistic. **Figure S3**. The distribution of number of accessions occurred gSNP sites. **Figure S4**. Distribution of minor haplotype frequency at all loci. **Figure S5**. The average divergence between A. lyrata and each haplotype of rare and intermediate alleles. **Figure S6**. The patterns of extension of rare-alleles in 9 loci. (DOCX 758 kb)

## Abbreviations

SNP: Single nucleotide polymorphism
Indel: Insertion/deletion
gSNP: Grouped single nucleotide polymorphism
rSNP: Random single nucleotide polymorphism
nfSNP: Non-fixed single nucleotide polymorphism
dSNP: Dispersed single nucleotide polymorphism
