# Supplementary material for: Genomic variations and distinct evolutionary rate of rare alleles in Arabidopsis thaliana
Source: BMC Evol Biol. 2016 Jan 27;16:25. doi: 10.1186/s12862-016-0590-7 (PMC4728917; doi:10.1186/s12862-016-0590-7)
Supplement: Additional file 1: Figure S1. — Results of coalescent simulations. Figure S2. The distribution of Tajima’s D statistic. Figure S3. The distribution of number of accessions occurred gSNP sites. Figure S4. Distribution of minor haplotype frequency at all loci. Figure S5. The average divergence between A. lyrata and each haplotype of rare and intermediate alleles. Figure S6. The patterns of extension of rare-alleles in 9 loci. (DOCX 758 kb) [file 12862_2016_590_MOESM1_ESM.docx]

**Additional files**

**Figure S1:** Results of coalescent simulations. In this simulation we considered the probability of a 500-bp locus containing a certain number of linked SNPs in low frequency (<10%) accessions. The simulation is based on a neutral model with constant population size, no recombination, panmixis, and infinite sites. We repeated the simulation 10000 times, using software developed by Hudson (2002). The mutation parameter (θ) is 2.9 [=S/939/
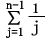
; S is the sum of SNPs obtained from the Nordborg dataset (Nordborg et al. 2005), the length of sequence is set as 500 base pairs and the accession number n is defined as 85, which is the same number as the average accession in 939 loci we used]. The black squares represent the number of fixed SNPs in a distinct haplotype which contained <10% frequency accessions (in total 85 accessions).

Notably, *P*_fixed SNPs≥5_ (the probability of a locus having 5 or more fixed SNPs in one distinct haplotype which contained less than 9 accessions) is <0.05 in 85 accessions, demonstrating that the rare alleles defined in Methods (which must have ≥5 fixed SNPs), are not random results.

**
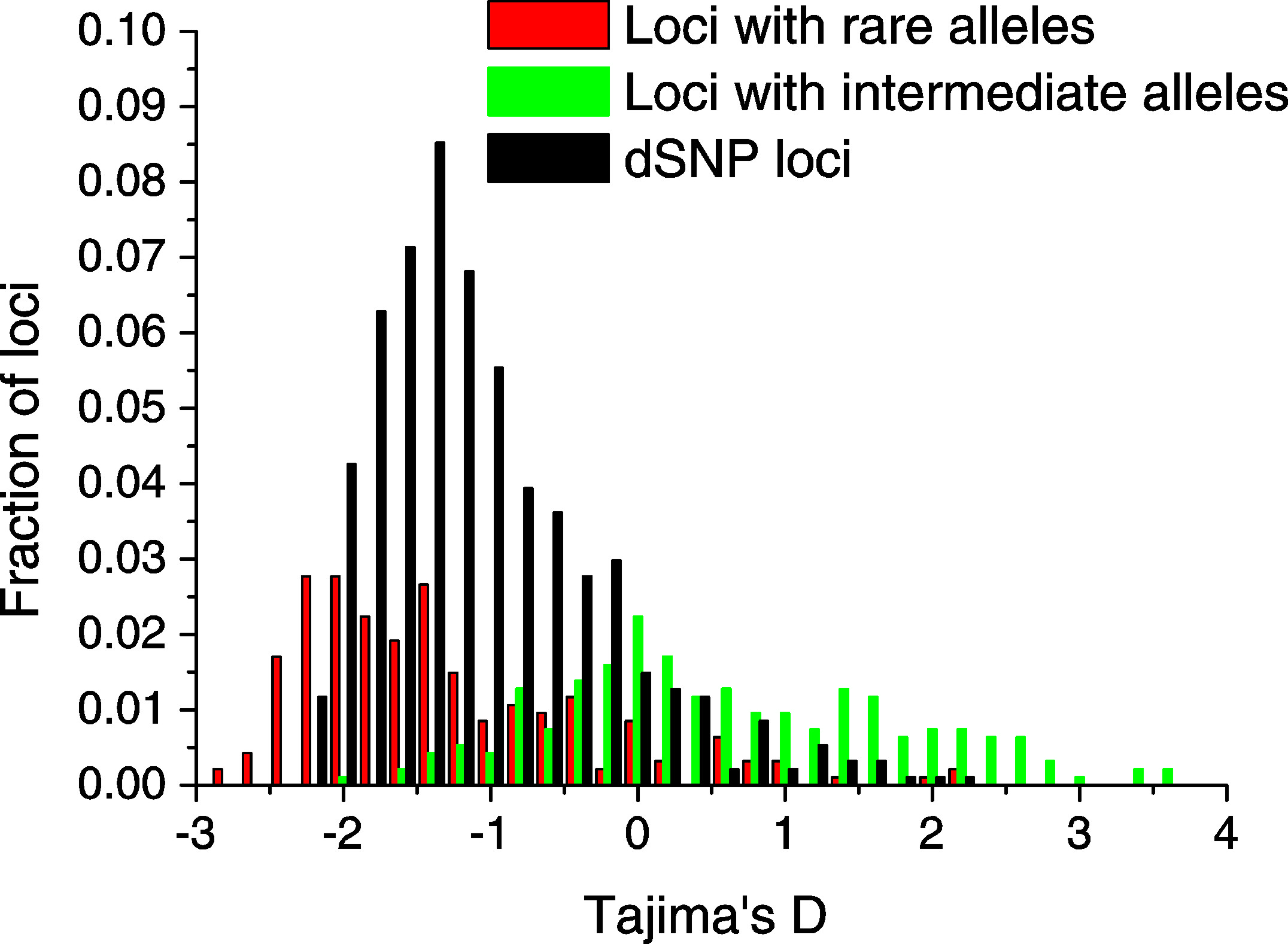
**

**Figure S2:** The distribution of Tajima’s D statistic for Rare allele, Intermediate allele, and dSNP loci, respectively.

**
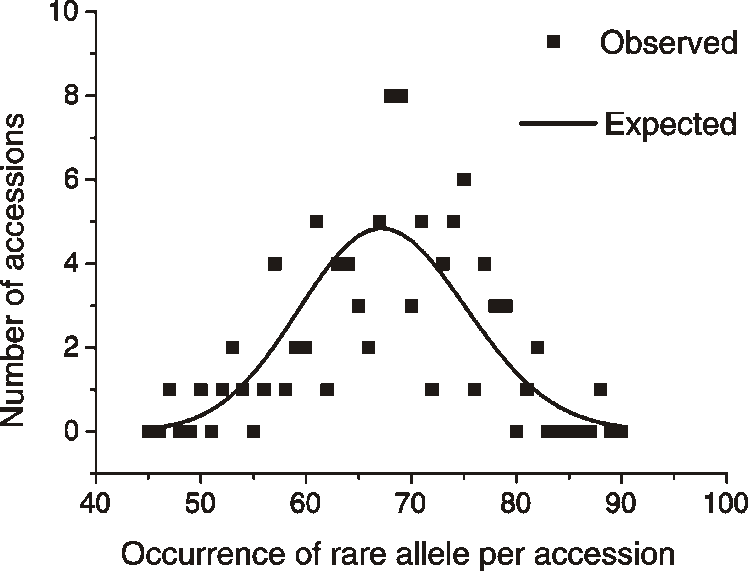
**

**Figure S3:** The distribution of number of accessions occurred gSNP sites in all loci (939), with expected number under random occurrence model of all the accessions.

**
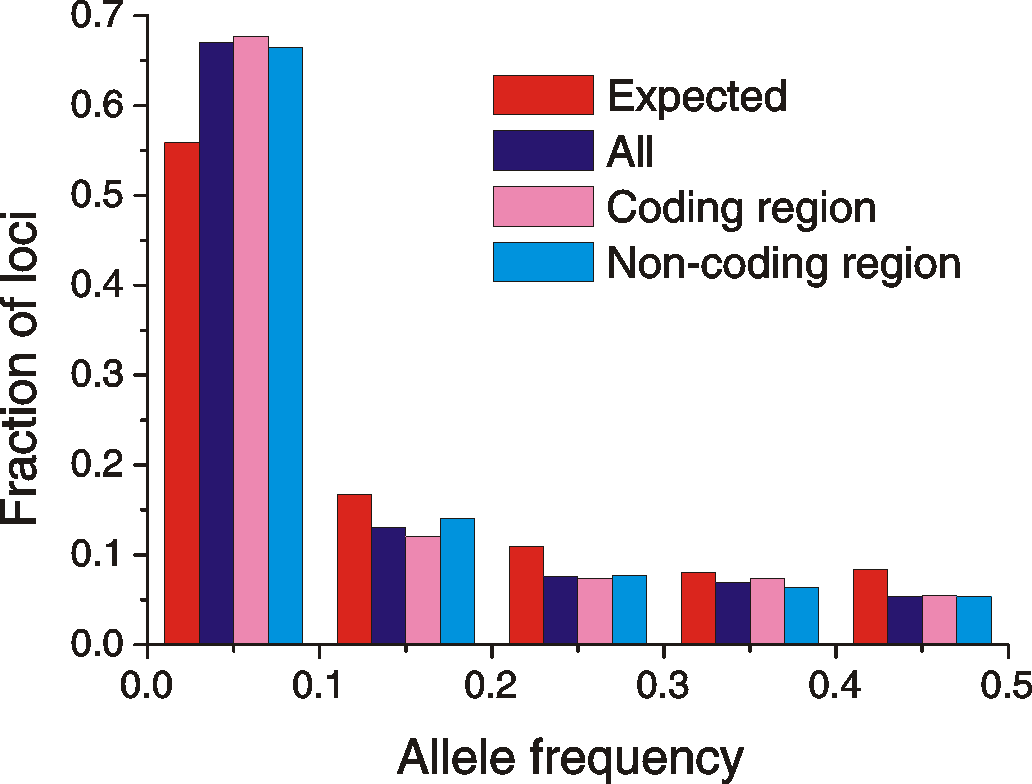
**

**Figure S4:** Distribution of minor haplotype frequency at all loci (939). Expected frequency is given by Tajima’s equation (1989), using a sample of average 87 individuals.


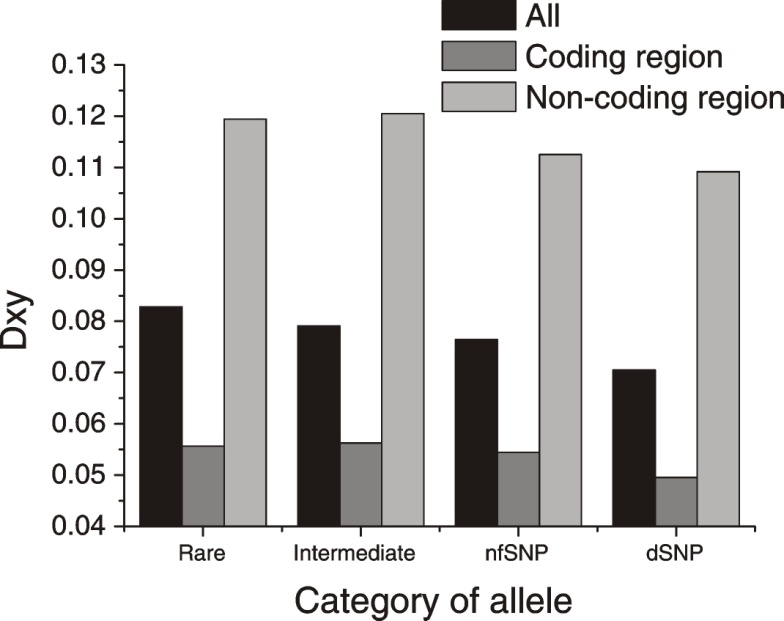


**Figure S5:** The average divergence between *A. lyrata* and each haplotype of rare and intermediate alleles in gSNP loci, and common alleles in gSNP and dSNP loci in all regions, coding and non-coding regions, respectively.

**
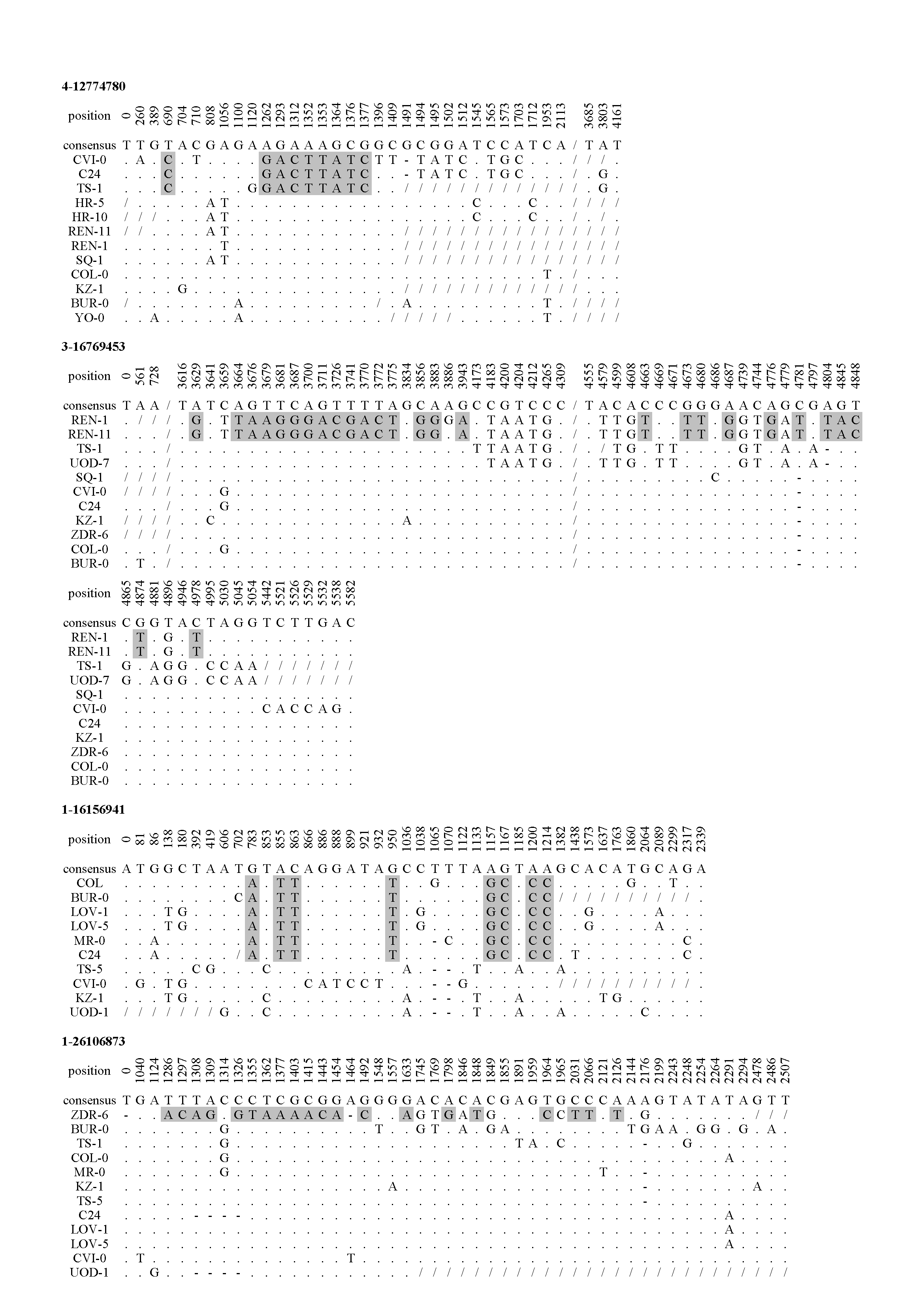
**

**
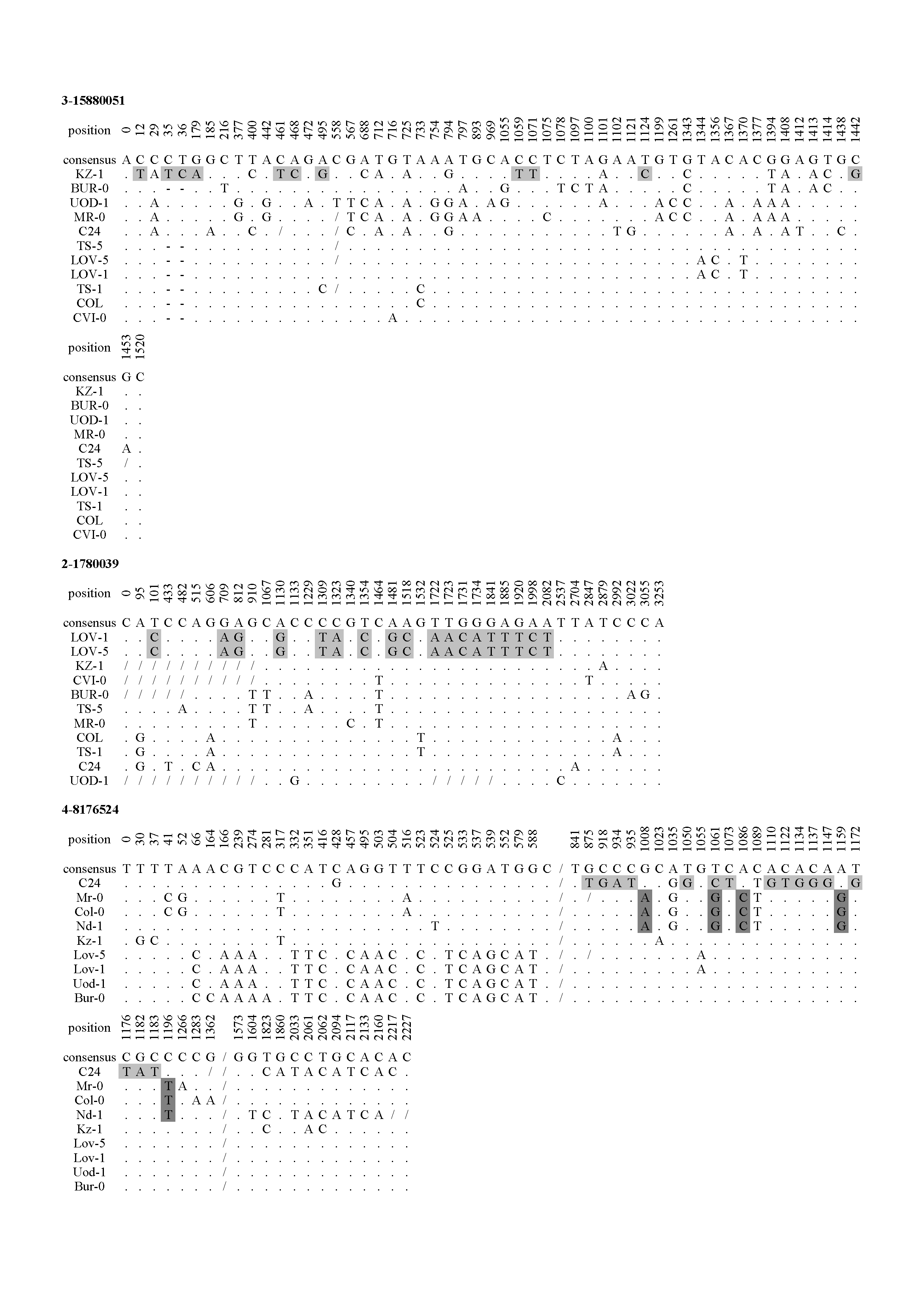
**

**
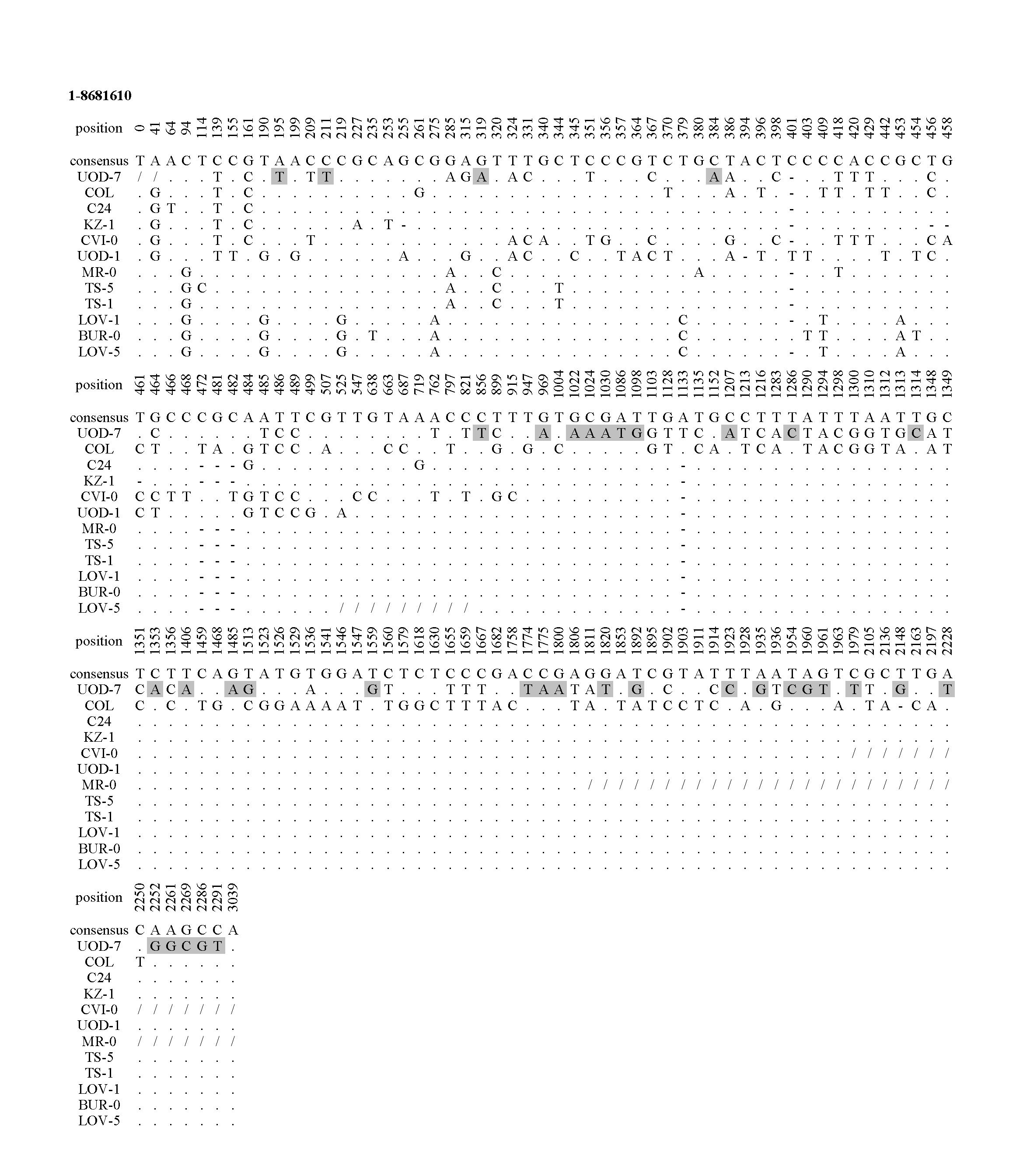
**

**
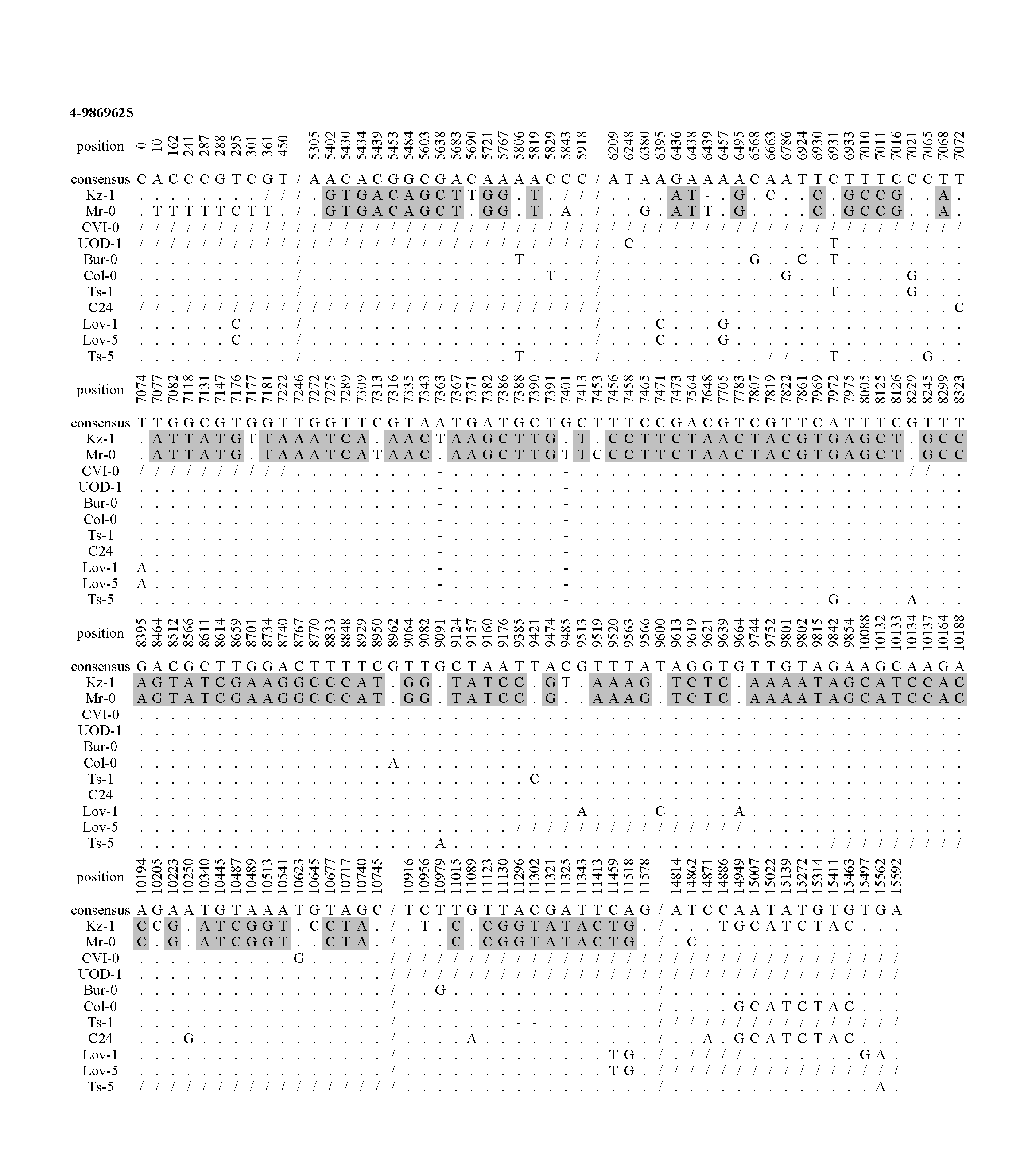
**

**Figure S6:** The patterns of extension of rare-alleles in 9 loci. The name of each locus is shown as the bold numbers, which denotes its location on chromosome with its start position (*A. thaliana* Version TAIR 8). For example, **4-9869625** means that it is located from 9869625 on chromosome 4. The numbers at the first line represents the relative position at each locus from start to end. The grey shadow denotes the pattern of rare haplotype. The dot in the figure represents the same nucleotide as the consensus, and ‘/’ refers to the ambiguous sequence due to the failure of PCR amplifications or sequencing reactions.
